# Supplementary material for: Low genetic diversity in captive populations of the critically endangered Blue-crowned Laughingthrush (Garrulax courtoisi) revealed by a panel of novel microsatellites
Source: PeerJ. 2019 Mar 20;7:e6643. doi: 10.7717/peerj.6643 (PMC6431135; doi:10.7717/peerj.6643)
Supplement: Supplemental Information 1 — Genetic kinship between each pair of individuals that has the highest likelihood at lower diagonal (PO = Parent / Offspring; FS = Full siblings; HS = Half siblings; U = Unrelated), and the pairwise relatedness based on the Queller and Goodnight estimator (RQG) are provided for each pair of individuals at upper diagonal. Table S2a. indicate OPHK population of Blue-crowned Laughingthrush, and Table S2b. indicate NCZ population of Blue-crowned Laughingthrush. (OPHK: Ocean Park Hong Kong, NCZ: Nanchang Zoo). [file peerj-07-6643-s001.docx]

**Table S1. Pairwise genetic relatedness among analysed pairs of Blue-crowned Laughingthrush individuals.**

Genetic kinship between each pair of individuals that has the highest likelihood at lower diagonal (PO = Parent / Offspring; FS = Full siblings; HS = Half siblings; U = Unrelated), and the pairwise relatedness based on the Queller and Goodnight estimator (R_QG_) are provided for each pair of individuals at upper diagonal. Table S2a. indicate OPHK population of Blue-crowned Laughingthrush, and Table S2b. indicate NCZ population of Blue-crowned Laughingthrush. (OPHK: Ocean Park Hong Kong, NCZ: Nanchang Zoo).

**Table S1a.**

| OPHK | 6027 | 6028 | 6029 | 6030 | 6031 | 6032 | 6033 | 6034 | 6035 | 6036 | 6037 | 6038 | 6039 | 6040 |
| --- | --- | --- | --- | --- | --- | --- | --- | --- | --- | --- | --- | --- | --- | --- |
| SYSb6027 |  | -0.58 | -1.09 | -0.46 | -0.38 | -0.69 | -0.65 | -0.41 | -0.51 | -0.38 | -0.52 | -0.88 | -1.05 | -1.05 |
| SYSb6028 | U |  | 0.15 | 0.67 | 0.07 | -0.56 | -0.30 | 0.62 | 0.10 | 0.07 | -0.84 | 0.14 | -0.70 | -0.70 |
| SYSb6029 | U | HS |  | -0.39 | -0.44 | -0.70 | -0.29 | -0.29 | -0.19 | -0.44 | -0.19 | -0.31 | -0.47 | -0.47 |
| SYSb6030 | U | PO | HS |  | -0.07 | -0.69 | -0.44 | 0.46 | -0.07 | -0.07 | -0.74 | -0.02 | -0.94 | -0.94 |
| SYSb6031 | U | U | U | U |  | 0.61 | 0.38 | 0.21 | 0.81 | 1.00 | 0.18 | 0.37 | 0.42 | 0.42 |
| SYSb6032 | U | U | U | U | U |  | -0.12 | -0.44 | 0.17 | 0.61 | 0.47 | -0.13 | 0.71 | 0.71 |
| SYSb6033 | U | U | U | U | U | U |  | 0.31 | 0.18 | 0.38 | -0.03 | 0.45 | -0.44 | -0.44 |
| SYSb6034 | U | FS | U | HS | U | U | PO |  | 0.28 | 0.21 | -0.55 | 0.31 | -0.47 | -0.47 |
| SYSb6035 | U | U | U | U | U | PO | U | U |  | 0.81 | 0.21 | 0.17 | 0.45 | 0.45 |
| SYSb6036 | U | U | U | U | FS | FS | U | U | PO |  | 0.18 | 0.37 | 0.42 | 0.42 |
| SYSb6037 | U | U | HS | U | U | PO | U | U | PO | U |  | -0.56 | 0.76 | 0.76 |
| SYSb6038 | U | HS | U | U | U | U | HS | U | U | HS | U |  | -0.45 | -0.45 |
| SYSb6039 | U | U | FS | U | U | FS | U | U | FS | U | PO | U |  | 1.00 |
| SYSb6040 | U | U | HS | U | U | FS | U | U | PO | U | FS | U | FS |  |

**Table S1b.**

| NCZ | 6041 | 6042 | 6043 | 6044 | 6045 | 6046 | 6047 | 6048 | 6049 |
| --- | --- | --- | --- | --- | --- | --- | --- | --- | --- |
| SYSb6041 |  | -0.48 | 0.32 | -0.24 | -0.13 | -0.17 | -0.28 | 0.08 | -0.24 |
| SYSb6042 | U |  | 0.46 | 0.61 | -0.07 | -0.54 | -0.41 | -0.31 | -0.02 |
| SYSb6043 | U | HS |  | -0.13 | -0.04 | -0.38 | -0.40 | -0.11 | 0.12 |
| SYSb6044 | U | PO | U |  | -0.02 | 0.18 | -0.01 | -0.30 | -0.31 |
| SYSb6045 | U | U | U | U |  | 0.07 | -0.07 | -0.03 | -0.92 |
| SYSb6046 | U | U | U | PO | U |  | 0.46 | -0.26 | -0.27 |
| SYSb6047 | U | U | U | U | U | HS |  | -0.60 | 0.12 |
| SYSb6048 | U | U | U | U | U | U | U |  | -0.27 |
| SYSb6049 | U | U | U | U | U | U | FS | U |  |
