# Supplementary material for: Low genetic diversity in captive populations of the critically endangered Blue-crowned Laughingthrush (Garrulax courtoisi) revealed by a panel of novel microsatellites
Source: PeerJ. 2019 Mar 20;7:e6643. doi: 10.7717/peerj.6643 (PMC6431135; doi:10.7717/peerj.6643)
Supplement: Supplemental Information 2 — Raw Data. Abbreviations indicate different Blue-crowned Laughingthrush populations (OPHK: Ocean Park Hong Kong, NCZ: Nanchang Zoo). [file peerj-07-6643-s002.docx]

**Table S2. Genotype of the 12 microsatellite loci in a sample set of two captive Blue-crowned Laughingthrush populations.**

Abbreviations indicate different Blue-crowned Laughingthrush populations (OPHK: Ocean Park Hong Kong, NCZ: Nanchang Zoo).

| Sample ID | Population ID | BCLT_L1 | BCLT_L2 | BCLT_L3 | BCLT_L4 | BCLT_L5 | BCLT_L6 | BCLT_L7 | BCLT_L8 | BCLT_L9 | BCLT_L10 | BCLT_L11 | BCLT_L12 |
| --- | --- | --- | --- | --- | --- | --- | --- | --- | --- | --- | --- | --- | --- |
| SYSb6027 | OPHK | 106 | 102 | 108 | 124 | 122 | 192 | 223 | 246 | 244 | 143 | 188 | 263 |
|  | OPHK | 108 | 104 | 108 | 124 | 136 | 192 | 223 | 248 | 244 | 143 | 188 | 263 |
| SYSb6028 | OPHK | 105 | 104 | 108 | 124 | 124 | 192 | 225 | 246 | 244 | 143 | 182 | 263 |
|  | OPHK | 106 | 104 | 108 | 124 | 138 | 192 | 225 | 248 | 244 | 143 | 188 | 266 |
| SYSb6029 | OPHK | 105 | 104 | 108 | 124 | 122 | 192 | 223 | 248 | 244 | 143 | 182 | 263 |
|  | OPHK | 106 | 104 | 108 | 126 | 124 | 194 | 225 | 248 | 246 | 146 | 188 | 263 |
| SYSb6030 | OPHK | 105 | 104 | 108 | 124 | 138 | 190 | 223 | 248 | 244 | 143 | 182 | 263 |
|  | OPHK | 106 | 104 | 108 | 124 | 138 | 192 | 225 | 248 | 244 | 146 | 188 | 263 |
| SYSb6031 | OPHK | 106 | 104 | 112 | 124 | 136 | 192 | 225 | 248 | 244 | 143 | 188 | 263 |
|  | OPHK | 106 | 104 | 112 | 124 | 136 | 194 | 225 | 248 | 244 | 146 | 188 | 263 |
| SYSb6032 | OPHK | 106 | 104 | 108 | 124 | 136 | 192 | 223 | 248 | 242 | 143 | 188 | 263 |
|  | OPHK | 106 | 104 | 108 | 126 | 136 | 194 | 225 | 248 | 244 | 146 | 188 | 263 |
| SYSb6033 | OPHK | 106 | 104 | 108 | 124 | 124 | 194 | 225 | 248 | 244 | 143 | 188 | 263 |
|  | OPHK | 106 | 104 | 108 | 124 | 136 | 196 | 225 | 248 | 246 | 143 | 188 | 266 |
| SYSb6034 | OPHK | 106 | 104 | 108 | 124 | 124 | 192 | 225 | 248 | 244 | 143 | 188 | 263 |
|  | OPHK | 106 | 104 | 108 | 124 | 138 | 196 | 225 | 248 | 244 | 143 | 188 | 266 |
| SYSb6035 | OPHK | 106 | 104 | 108 | 124 | 122 | 192 | 223 | 248 | 244 | 146 | 188 | 263 |
|  | OPHK | 106 | 104 | 108 | 124 | 136 | 194 | 225 | 248 | 244 | 146 | 188 | 263 |
| SYSb6036 | OPHK | 106 | 104 | 108 | 124 | 136 | 192 | 225 | 248 | 244 | 143 | 188 | 263 |
|  | OPHK | 106 | 104 | 108 | 124 | 136 | 194 | 225 | 248 | 244 | 146 | 188 | 263 |
| SYSb6037 | OPHK | 106 | 104 | 108 | 124 | 122 | 194 | 223 | 248 | 242 | 146 | 188 | 263 |
|  | OPHK | 106 | 104 | 108 | 126 | 136 | 194 | 223 | 248 | 244 | 146 | 188 | 263 |
| SYSb6038 | OPHK | 106 | 104 | 108 | 124 | 124 | 190 | 225 | 246 | 244 | 143 | 188 | 263 |
|  | OPHK | 106 | 104 | 108 | 124 | 136 | 192 | 225 | 248 | 246 | 146 | 188 | 263 |
| SYSb6039 | OPHK | 106 | 104 | 108 | 124 | 122 | 192 | 223 | 248 | 242 | 143 | 188 | 263 |
|  | OPHK | 106 | 104 | 108 | 126 | 136 | 194 | 225 | 248 | 244 | 146 | 188 | 263 |
| SYSb6040 | OPHK | 106 | 104 | 108 | 124 | 122 | 192 | 223 | 248 | 242 | 143 | 188 | 263 |
|  | OPHK | 106 | 104 | 108 | 126 | 136 | 194 | 223 | 248 | 244 | 146 | 188 | 263 |
| SYSb6041 | NCZ | 106 | 104 | 108 | 124 | 122 | 192 | 223 | 246 | 244 | 140 | 182 | 257 |
|  | NCZ | 106 | 106 | 108 | 124 | 138 | 194 | 225 | 248 | 244 | 146 | 188 | 263 |
| SYSb6042 | NCZ | 106 | 104 | 108 | 122 | 122 | 196 | 223 | 248 | 241 | 143 | 188 | 263 |
|  | NCZ | 106 | 104 | 112 | 124 | 138 | 196 | 225 | 248 | 243 | 143 | 188 | 263 |
| SYSb6043 | NCZ | 106 | 104 | 108 | 124 | 138 | 192 | 225 | 248 | 244 | 143 | 182 | 263 |
|  | NCZ | 108 | 104 | 108 | 124 | 138 | 196 | 225 | 248 | 244 | 143 | 188 | 263 |
| SYSb6044 | NCZ | 106 | 104 | 108 | 124 | 122 | 196 | 223 | 248 | 242 | 143 | 188 | 263 |
|  | NCZ | 106 | 104 | 112 | 124 | 138 | 196 | 223 | 248 | 244 | 146 | 188 | 263 |
| SYSb6045 | NCZ | 106 | 104 | 108 | 122 | 122 | 196 | 225 | 246 | 244 | 146 | 188 | 263 |
|  | NCZ | 106 | 104 | 110 | 124 | 138 | 196 | 227 | 248 | 246 | 146 | 188 | 263 |
| SYSb6046 | NCZ | 106 | 104 | 108 | 124 | 124 | 192 | 223 | 248 | 242 | 146 | 188 | 263 |
|  | NCZ | 106 | 104 | 112 | 124 | 138 | 196 | 227 | 248 | 244 | 146 | 188 | 263 |
| SYSb6047 | NCZ | 106 | 104 | 108 | 122 | 124 | 196 | 223 | 246 | 242 | 146 | 188 | 263 |
|  | NCZ | 106 | 104 | 112 | 124 | 124 | 202 | 225 | 248 | 244 | 146 | 188 | 263 |
| SYSb6048 | NCZ | 106 | 104 | 108 | 124 | 122 | 192 | 223 | 248 | 246 | 143 | 182 | 263 |
|  | NCZ | 108 | 104 | 108 | 124 | 138 | 194 | 227 | 248 | 246 | 146 | 188 | 263 |
| SYSb6049 | NCZ | 106 | 104 | 108 | 122 | 124 | 192 | 223 | 246 | 242 | 143 | 188 | 263 |
|  | NCZ | 106 | 104 | 112 | 124 | 124 | 194 | 225 | 248 | 244 | 143 | 188 | 263 |
