# Supplementary figures and images for: Low genetic diversity in captive populations of the critically endangered Blue-crowned Laughingthrush (Garrulax courtoisi) revealed by a panel of novel microsatellites
Source: PeerJ. 2019 Mar 20;7:e6643. doi: 10.7717/peerj.6643 (PMC6431135; doi:10.7717/peerj.6643)

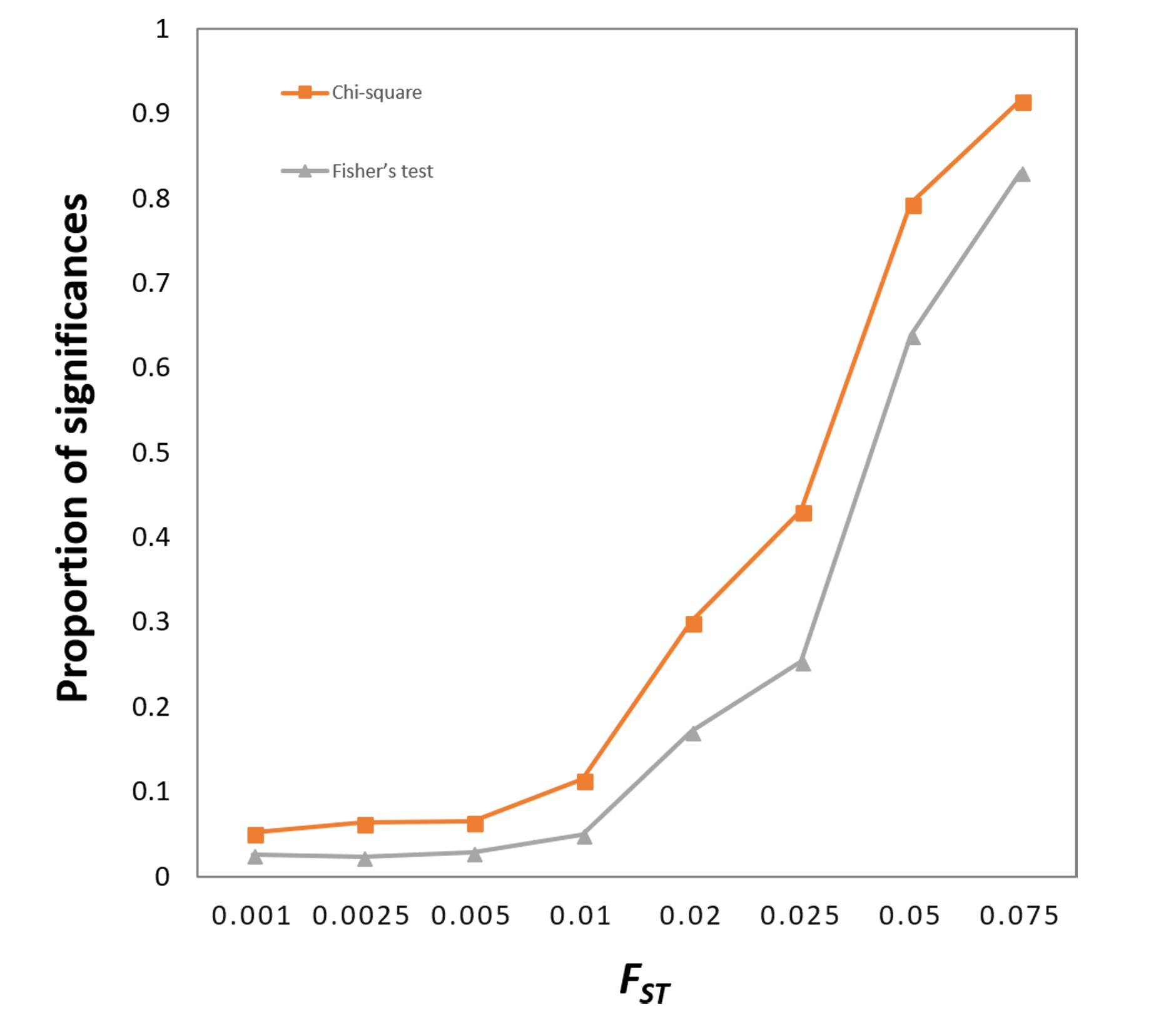

Supplement: Supplemental Information 3 — Datasets with eight predefined levels of population differentiation (FST values of 0.001, 0.0025, 0.005, 0.01, 0.02, 0.05, 0.075) were generated using POWSIM. Statistical power was defined as the proportion of times the null hypothesis of equal allele frequencies across populations was rejected using a chi-square test or a Fisher’s exact test. [file peerj-07-6643-s003.png]
